# Supplementary figures and images for: Aspirin-triggered resolvin D1 reduces parasitic cardiac load by decreasing inflammation in a murine model of early chronic Chagas disease
Source: PLoS Negl Trop Dis. 2021 Nov 16;15(11):e0009978. doi: 10.1371/journal.pntd.0009978 (PMC8631674; doi:10.1371/journal.pntd.0009978)

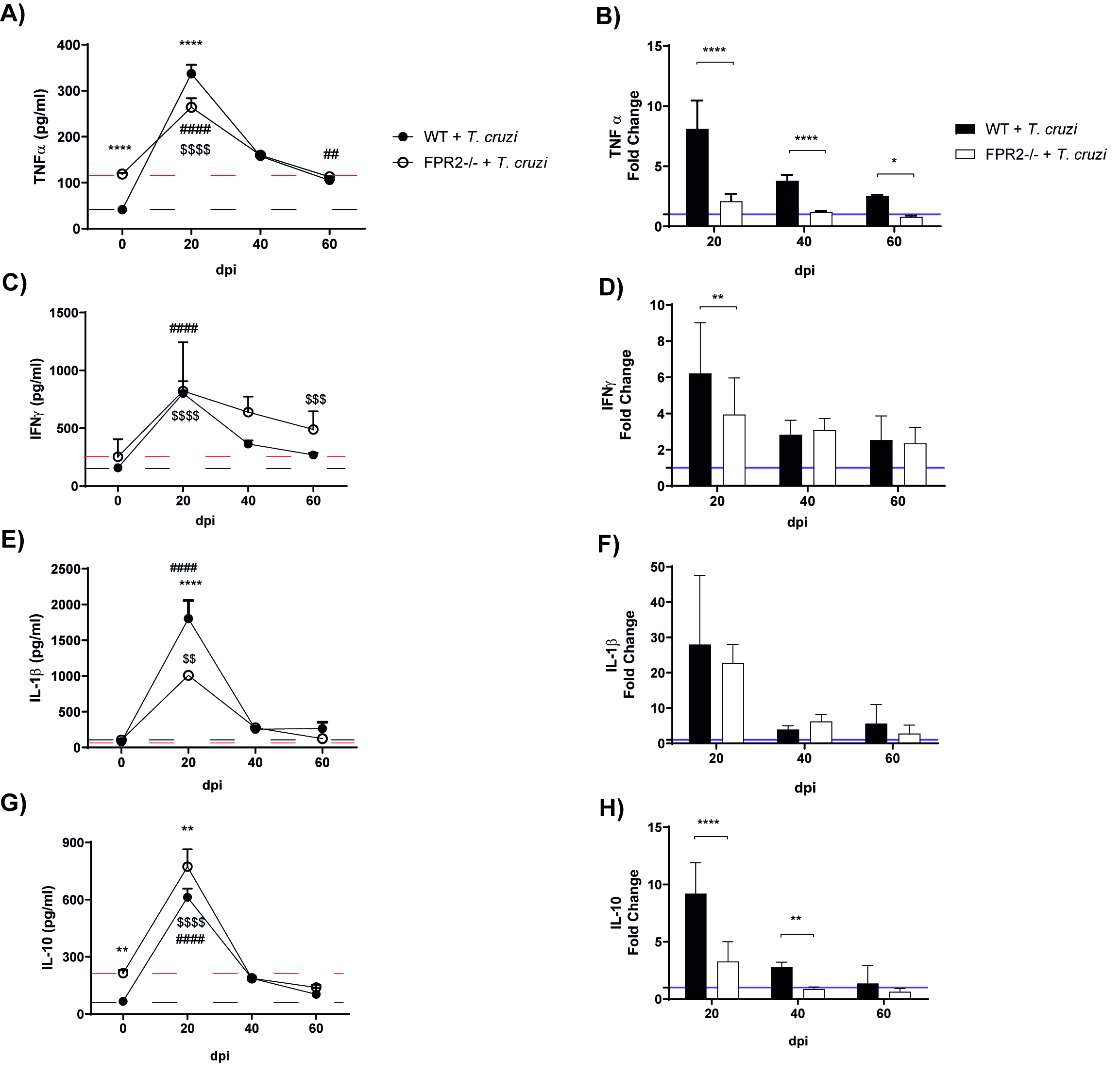

Supplement: S1 Fig — The concentrations of TNFα (A-B), IFNγ (C-D), IL-1β (E-F), and IL-10 (G-H) were quantified in the serum from C57BL/6 mice uninfected and infected with T. cruzi at 20, 40, and 60 dpi, using ELISA assays. Data are expressed as the mean ± SEM from one experiment (n = 8 mice per group). Two-way ANOVAs and Tukey’s post-hoc tests were performed to identify significant differences. Asterisks indicate significant differences between infected WT and infected FPR2. Dollar signs indicate significant differences between healthy FPR2-/- and infected FPR2-/-. Number signs indicate significant differences between healthy WT and infected WT. one symbol, p ≤ 0.05; two symbols, p ≤ 0.01; three symbols, p ≤ 0.001; four symbols, p ≤ 0.0001. dpi, days post-infection. (TIF) [file pntd.0009978.s001.tif]

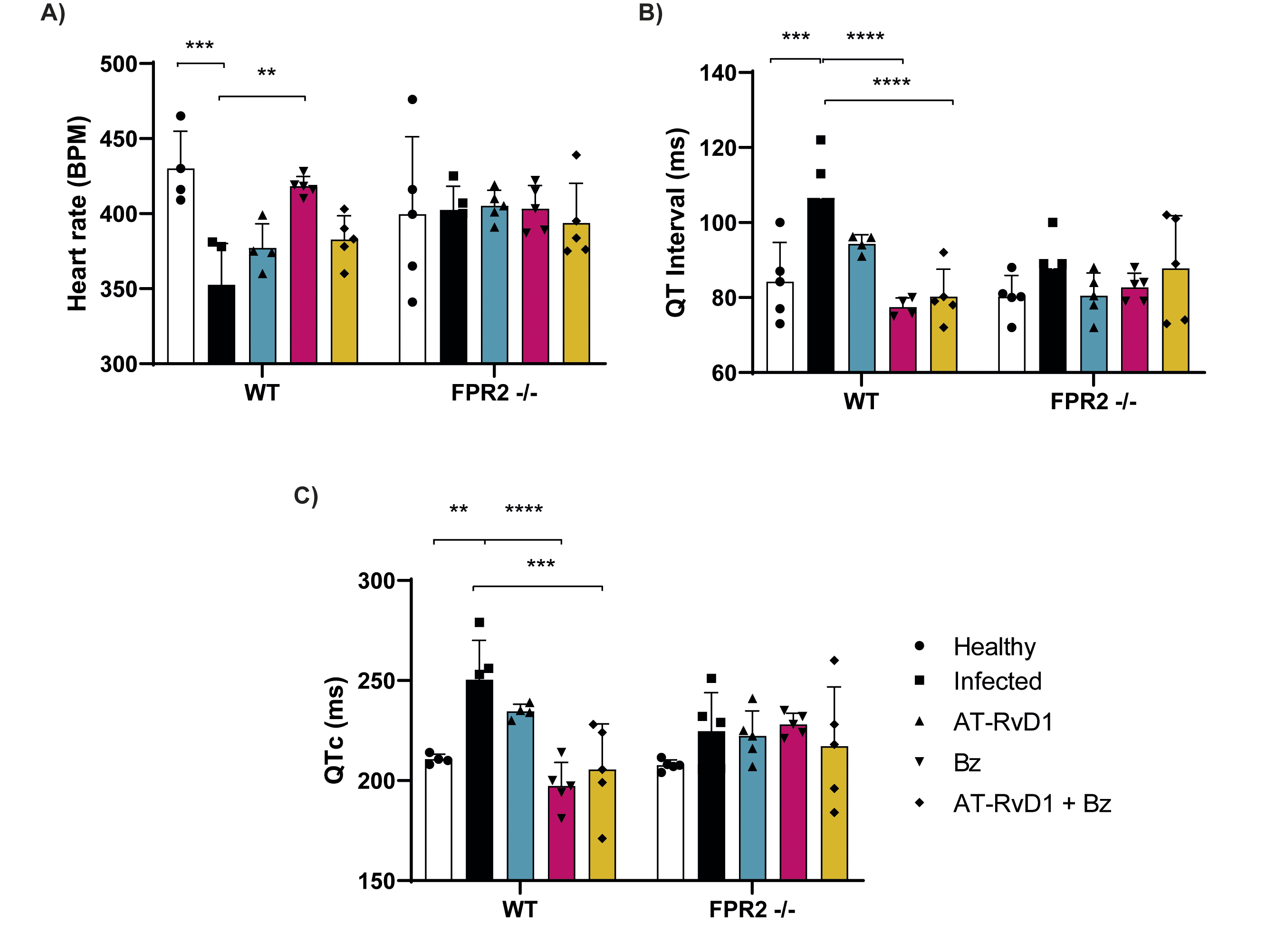

Supplement: S2 Fig — The variation in heart rate (A), QT Interval (B), and QTc (C) are presented. The statistical analysis used was two-way ANOVA followed by Tukey’s post-hoc tests (n = 8 mice per group). **p ≤ 0.01, ***p ≤ 0.001, ****p ≤ 0.0001. QTc, corrected QT interval. (TIF) [file pntd.0009978.s002.tif]

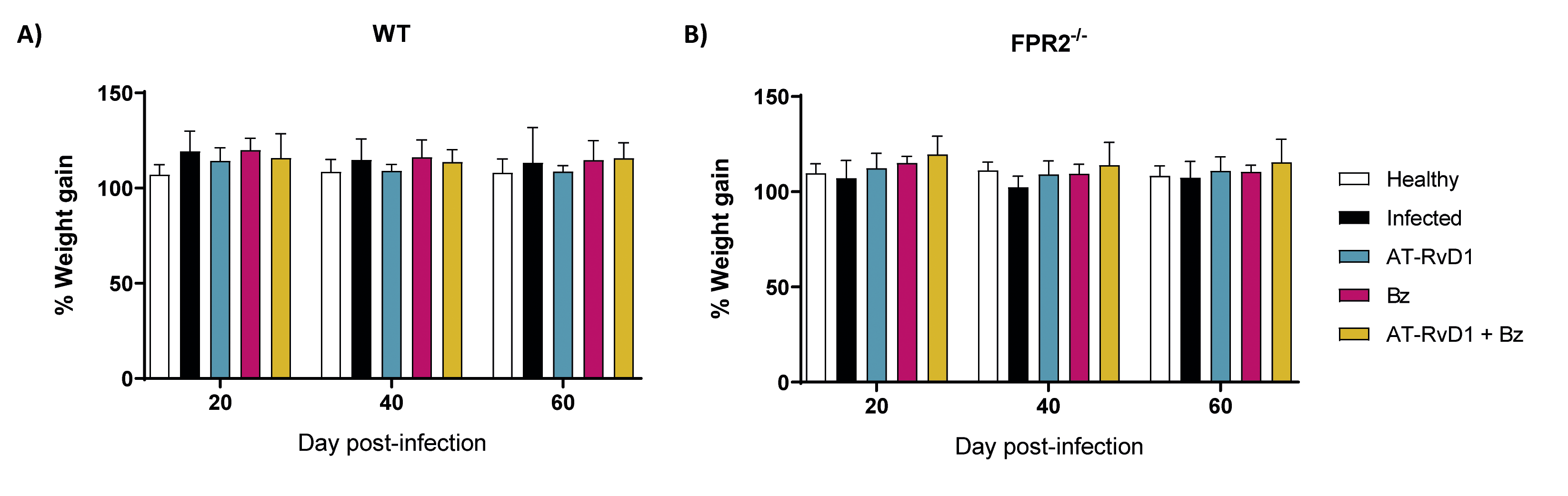

Supplement: S3 Fig — Mice were infected with T. cruzi (Dm28) trypomastigotes and treated with 5 μg/Kg//day AT-RvD1 or 30 mg/kg/day Bz between day 40 and 60 postinfection. For the combination, Bz dose was 5 mg/kg/day (n = 8 mice per group) (TIF) [file pntd.0009978.s003.tif]
