# Supplementary material for: Aspirin-triggered resolvin D1 reduces parasitic cardiac load by decreasing inflammation in a murine model of early chronic Chagas disease
Source: PLoS Negl Trop Dis. 2021 Nov 16;15(11):e0009978. doi: 10.1371/journal.pntd.0009978 (PMC8631674; doi:10.1371/journal.pntd.0009978)
Supplement: Attachment — Submitted filename: Response to Reviewers.docx [file pntd.0009978.s004.docx]

**Response to reviewers:**

**We are grateful to the reviewers for their thoughtful and accurate comments, insights and suggestions, which have undoubtedly contributed to a substantial improvement in the quality of the present work. We hope that our responses will be favorably received and that the manuscript will now be suitable for publication in Plos Neglected Tropical Diseases.**

**The manuscript has been rechecked and the necessary changes have been made in accordance with the reviewers’ suggestions. The responses to reviewers’ comments are included below:**

**Reviewer #2:**

The study goal is sufficiently articulated with a testable hypothesis. The authors attempt to demonstrate that the resolution of inflammation is valuable as a pharmacological strategy for chronic Chagas heart disease.
Using a murine model, they evaluate administration of AT-RvD1, a pro-resolving lipid mediator effective against inflammation, to dampen pathological inflammatory responses and achieve parasite elimination. Thus, the study design is appropriate to address the stated objective.

The animal protocol is clearly described and the number of mice included for the different experiments allows validation of the hypothesis being tested.
Statistical analyses used give support to the authors’ conclusions.
No concerns about ethical or regulatory requirements have been identified.

Specific comments:

The experimental model is based on C57BL/6 mice at 60 days of infection with T. cruzi strain Dm28c (indicate TcI lineage). This reflects the beginning of prolonged infection, showing moderate inflammatoty pathology and lack of important ECG abnormalities. Therefore, the study title should be changed to “…in a murine model of early chronic Chagas disease”.

Thank you very much for this comment. We agree that the observational period is not congruous with an advanced chronic stage. Accordingly, the title has been changed as suggested, and this concern has been addressed throughout the manuscript, wherever appropriate (marked in green letters for easy tracking), and further discussed in the discussion section (Please see lines 607-613 in the revised manuscript).

As per your suggestion, the appropriate discrete typing unit DTU of the Dm28 strain has been indicated.

On what basis have the authors chosen the dose and duration of treatment with AT-RvD1?

The duration of the treatment was chosen based on a method that used 15-epi-LxA2 and was published by our team (Antimicrob Agents Chemother. 2017 Feb 23;61: e02137-16; reference included in the revised manuscript). Although the resolving lipid is different in both the studies, both resolvin, used in the present study, and simvastatin, used in the previous study, are triggered by aspirin and possess greater stability than their physiological counterparts.

We used 5 μg/kg of AT-RvD1, which is slightly higher than that used by Horta et al (3 μg/Kg). This was done based on the understanding that the administered dose is equivalent to approximately 100 ng/animal, which is within the effective range of doses reported by Sun et al. for decreasing peritoneal leukocyte infiltration (Sun YP, Oh SF, Uddin J, Yang R, Gotlinger K, Campbell E, et al. Resolvin D1 and its aspirin-triggered 17R epimer. Stereochemical assignments, anti-inflammatory properties, and enzymatic inactivation. J Biol Chem. 2007;282: 9323-9334). The latter reference has been included in the revised manuscript.

As per your comment, this concern has been clarified in the methodology section. Please see line 182.

Please indicate the number of independent experiments performed for each figure.

For ethical reasons, only one cohort of mice was evaluated to reduce the number of animals. The estimated statistical power was sufficient, considering that each animal contributed to an independent experiment. The number of mice evaluated (n) per experiment has been specified in each figure. Detailed information has been included in the methodology section of the revised manuscript (lines 180 and 186).

Reviewer #2: The analysis presented relatively matches the original aim. The authors state that AT-RvD1 therapy is helpful to revert T. cruzi-elicited proinflammatory cytokine imbalance, mainly via FPR2 (lines 480-482). However, mRNA analysis of heart tissues reveals no significant reduction in myocardial IFNgamma and IL-1beta levels (Fig 3 B,C). Such paragraph should be re-written.

Thank you very much for this observation.

We agree that there is an obvious discrepancy between plasma cytokine levels and the corresponding mRNA levels in the heart. The imbalance that you have mentioned refers mainly to the findings in plasma, where the influence of FPR2 on proinflammatory cytokines is clearer. However, the lack of correlation with mRNA levels in cardiac tissue is striking, and we do not have an explanation for this discrepancy at the moment. Therefore, we have modified the sentence in which we stated that FPR2 plays a pivotal role in the anti-inflammatory effect. In addition, we have included a sentence about the possible involvement of GPR32. Therefore, as per your suggestion, this paragraph has been modified. Please see lines 531-535.

Moreover, AT-RvD1 demonstrated anti-hypertrophic effect through FPR2 in myocardium, but most beneficial actions (decrease in collagen deposition and production of natriuretic peptides; IL-10 overexpression) are independent of interaction with this receptor. Why the study title refers particularly to FPR2?

We appreciate this suggestion. The central objective of our study was to evaluate the participation of the FPR2 receptor in the anti-inflammatory effect of AT-Rv-D1. However, we agree with you that the observed effects cannot be attributed exclusively to FPR2 intermediation; therefore, the term “FPR2” has been removed from the title.

Additionally, the outcome of coadministration of AT-RvD1 and benznidazole is poorly analyzed. The effects of combination are often worst than those achieved with each individual drug. Does Bz affect AT-RvD1 activity when administered simultaneously? The current findings strongly discourage the potential use of AT-RvD1 + Bz combined therapy for Chagas. **A comment about this observation should be included in the Discussion section.**

We thank you for pointing this out. This is certainly an interesting situation to analyze. The administration of benznidazole and AT-RvD1 did occur simultaneously, as indicated in the methods section of the revised manuscript (line 187).

We agree that in many observations, the combination of benznidazole and AT-RvD1 has produced an adverse effect. Given the results of this study, this combination might have promoted a more proinflammatory scenario, contrary to what we sought to attain. Therefore, it may be more appropriate to suggest a scheme in which an inflammation-resolving agent is administered first, which also facilitates parasite elimination (perhaps by efferocytosis, as mentioned below), followed by a course of therapy with benzindazole, which could be shorter or a lower dose of the antiparasitic could be used to avoid adverse events. This concern has been addressed in the discussion. Please see lines 554-562 in the revised manuscript.

The overall presentation of results is correct, including figures of adequate quality.
Specific comments:

In a recent report by Horta and colleagues (Infect Immun 2020; 88:e00052-20), a decrease in body mass was recorded upon RvD1 treatment in chagasic mice. Have the authors observed such deleterious effect in their series?

We agree that this is undoubtedly a valuable observation, given the report by Horta et al. However, in our study, no effect of AT-RvD1 on body weight was observed throughout the experiment. Of note, body weight was measured according to the animal monitoring protocol. A figure has been included in the supplementary material addressing this issue, which is cited in the text. Please see lines 596-600.

Increased levels of myocardial TGFbeta is a hallmark of T. cruzi-triggered fibrosis. Have the authors any data of this cytokine in AT-RvD1-receiving mice?

We appreciate this insight. Evidently, TGFβ is an appropriate marker of fibrosis in the context of *T. cruzi* infection. However, in the present study, we used α-SMA as a marker of fibrosis, which correlated directly with collagen deposition. This isoform of actin, predominant in vascular smooth muscle, plays an important role in fibrogenesis and correlates with the presence of myofibroblasts, which play a crucial role in the fibrotic response. Importantly, α-SMA production is dependent on the TGFβ-differentiating action on fibroblasts. Furthermore, this observation aligns with the findings of a study by Coelho et al (Parasit Vectors. 2018 Jan 30;11(1):72). Accordingly, Figure 4 was modified to include this result. Moreover, the text in line 412 was modified to include this description, and it was also included in the discussion section (please, see lines 587-592).

Regarding the reduced parasite load in hearts from infected and AT-RvD1-treated mice and, besides, the enhancement of immune effector mechanisms, occurrence of pathogen clearance through efferocytosis should not be discarded, as described previously (Karaji & Sattentau, Front Immunol 2017; 8:1863). This could be commented on within the Discussion.

It is interesting to consider efferocytosis as a possible mechanism that explains the decrease in parasite load in the heart. The comment and the suggestion are appreciated. This concern was addressed in the discussion section in lines 623-626. However, since efferocytosis is a process that occurs in the presence of apoptotic bodies or dying cells, it would not be reasonable to presume that it is the only mechanism involved in this finding.

The alternative receptor for D1 resolvins is GPR 32 (reference 58 of the paper). Please correct line 514 of the text.

The suggested correction has been incorporated in line 596 of the revised manuscript.

Lines 483-485: in addition to NFkappaB inhibition, Gu and colleagues (Innate Immun 2016; 22:186-195) have shown a key role for GSK3beta and CREB in the anti-inflammatory actions of resolvin D1. This finding should be mentioned and the new reference added.

Indeed, this is an important observation, for which we are grateful. Consequently, this line of evidence has been incorporated into the discussion, and the suggested reference has been added. Please see lines 538-541 of the revised manuscript.

**Reviewer #2:** Most conclusions are supported by the data presented (see exceptions detailed above). Some aspects of analysis are limited by the experimental model chosen; e.g. ECG alterations should be better analyzed later in the course of infection.

The authors do discuss how these data can be helpful to advance our understanding of chronic Chagas heart disease and optimize specific chemotherapy. They clearly explain the worldwide relevance of Chagas cardiomyopathy for public health.

The limitations of the animal model used in this study and the consequences of the electrocardiographic analysis have been incorporated in a new paragraph (lines 607-613), as mentioned above.

**REVIEWER #3**

Reviewer #3: -Are the objectives of the study clearly articulated with a clear testable hypothesis stated? YES

-Is the study design appropriate to address the stated objectives? YES

-Is the population clearly described and appropriate for the hypothesis being tested? ANIMAL MODEL

-Is the sample size sufficient to ensure adequate power to address the hypothesis being tested? YES

-Were correct statistical analysis used to support conclusions? YES

-Are there concerns about ethical or regulatory requirements being met? YES

This paper studies the immunomodulatory experimental effects of AT-RvD1 and BZ treatment in a murine model of Chagas disease, isolated and in combination. The most relevant results are the regulation of the inflammatory response both at the systemic level and in the cardiac tissue, and the reduction in cellular infiltrates, cardiomyocyte hypertrophy, fibrosis, and the parasite load in the heart tissue. The work deserves to be published, but some important issues and limitations should be addressed, especially the following concepts that need to be revised:

Line 35: Both in the “Abstract” and in the “Author Summary” the sentences “After years of infection and in the absence of treatment, the disease progresses from an acute and asymptomatic phase to a chronic inflammatory cardiomyopathy, leading to heart failure and death” and “If the parasite is left untreated, the disease progresses from an acute symptomless phase to chronic 60 myocardial inflammation, which can cause heart failure and death years after infection.” should change to mention the percentage of patients progressing to CCC (30%). In its present form it transmits a misconception that all patients evolve to HF and death, which causes stigma and fear to CD affected people

Thank you very much for this suggestion. We agree that the reader may misinterpret the text. The corresponding sentences have been revised accordingly. (Please see lines 37 and 62 in the revised version)

Line 83- reference in a different format – WHO 2018; reference [1] is WHO 2002

The format of the reference has been updated in the revised manuscript.

Line 87 – please insert the reference related to 1% of patients treated

It has been estimated that a low percentage of patients seek treatment because the infection remains unnoticed during the acute phase and there are no suggestive signs or symptoms in most patients during the chronic phase. It is possible that this percentage is an underestimate of the actual number of patients obtaining treatment, due to active detection of infection at blood banks and in pregnant women in endemic countries. Therefore, we have deleted the sentence that stated that 1% of patients obtained treatment.

Line 91 – Sudden death is NOT the leading cause of mortality in patients with CCC, nor this idea is proposed in reference [5]

Thank you for this observation. Undoubtedly, the reference mentioned in your comment does not address the issue of sudden death. In a previous study (Circulation. 2018 Sep 18;138: e169-e209. doi: 10.1161/CIR.0000000000000599), the authors mentioned that sudden death occurs in 55%-60% of patients. As per your suggestion, this reference has been updated and the sentence has been modified to improve the accuracy of the statement. Please see line 93 in the revised manuscript.

Line 95: “The current treatment for CD utilizes nitroheterocyclic drugs, such as benznidazole (Bz), which are far from efficacious. This sentence shows no reference, and this is a concept that is under debate, specially under the results published after comparison of CCC progression rate by BZ treated and non-treated patients in a long-term follow-up cohort (Benznidazole decreases the risk of chronic Chagas disease progression and cardiovascular events: A long-term follow up study - https://doi.org/10.1016/j.eclinm.2020.100694) and the Samitrop study (Beneficial effects of benznidazole in Chagas disease: NIH SaMi-Trop cohort study - https://doi.org/10.1371/journal.pntd.0006814). This idea is repeated in the conclusion and should be changed or better contextualized in the manuscript.

We appreciate your comment. Certainly, the antiparasitic efficacy of benznidazole in both acute and chronic phases is undoubted, as it prevents significant cardiac alterations. However, uncertainty about the efficacy of benznidazole treatment in the chronic phase still persists, especially in situations where the chronic phase is more advanced. We agree that the recent body of evidence corroborates the efficacy of the current antiparasitic treatment, including the two references suggested by the reviewer, which have been incorporated into the manuscript. However, the adverse reactions caused by the usage of this drug may eventually decrease patient adherence or compliance to therapy and in severe cases, may cause discontinuation of treatment. This explains the current intense search for alternatives. Please see lines 98-101 in the revised manuscript.

This issue has also been addressed in the conclusion as per your suggestion. Please see lines 627-629.

Line 100: “The resolution of inflammation is an active process orchestrated by molecules known as specialized pro-resolving mediators (SPM) that culminate inflammatory processes [8]”. The verb “culminate” does not fit with the idea that authors seem wanting to propose.

Thank you very much for the correction. The term has been replaced as suggested. Please see line 106.

Line 154 – The animal model presents a limitation to be considered as a chronic model for human CCC by day 60 post infection. Two months is too soon to be considered as a chronic model, especially in C57Bl6 mice that are more resistant that Balb/C, CD1 or Swiss mice. Authors should have waited at least 90 -100 days to consider it a chronic model. This might explain why ECG alterations were not detected, as well as significant fibrosis in the WT model.

We agree with both the reviewers who pointed out this aspect of the study. The title has been modified to address this concern, and it has been specified throughout the text that the study was conducted in the early stage of the chronic phase, with the changes marked in green letters in the revised manuscript. Furthermore, this situation has also been addressed in the discussion section of the revised manuscript. Evidently, an evaluation of cardiac functions, especially electrocardiographic changes, early in the chronic phase may not show significant changes, as is probably the case in the present study, possibly contributing to a weakness in the results obtained. However, the reviewer would agree that the effect of AT-RvD1 on the pathophysiological events occurring at this stage of the disease, as seen in the changes in hypertrophy and fibrosis, as well as in the levels of biochemical markers of cardiac function, suggests the potential usefulness of host factor modifications in the treatment of Chagas disease, especially in the prevention of chronic deterioration arising from inflammatory effects. This concern has been addressed in lines 607-613 in the revised manuscript.

Please inform the number of animals used in each experiment and how many times the experiment was replicated. In the legend of the figures sometimes it is mentioned n=5 or n=8. The results do express a mean of n mice in one experiment or n mice in x experiments?

Thank you for this remark. Reviewer 2 has also pointed this out. The number of animals (eight) per group has been specified in the methodology section and in the respective figure legends of the revised manuscript. Please see line 180 and figure legends.

Besides, the authors did not mention if they used male or female mice, or both, a condition that also modulates host response.

Eight mice, four males and four females, were assigned to each treatment group, as stated in the methodology section (line 180). The untreated control group was composed of three male and two female mice. This information has been included in the revised manuscript (line 186). A homogeneous mixture of mice of both sexes was made under the premise that Chagas disease is not a condition that presents a sex preference. Although there may be gender-related aspects in host response, the susceptible population is homogeneous at this point of time, and so we intended to replicate it in the experimental design and received the approval of the bioethics committee.

Dm28c trypomastigotes less virulent than other strains and clones. These issues do not question the quality of the results obtained with the experiments presented but should be considered with reservations concerning interpretations and comparisons with human CCC.

Thank you very much for bringing this aspect of the study to our attention. This concern has been addressed in the discussion section, and a new paragraph has been included (lines 479-488). We agree that the virulence of the strain involved in the pathogenesis of Chagas disease is a crucial determinant of the progression of chronic phase. Given this, it is possible to find different strains in a patient due to different types of exposure. Furthermore, the distribution of strains and lineages varies regionally. Therefore, it would be unreasonable to extrapolate results obtained from a single strain to establish disease pathogenesis. However, it is logical to assume that strains in the TcI lineage may have similar behaviors. In any case, further studies are required. On the other hand, although Dm28 shows low virulence, the results pertaining to parasitemia and cardiac involvement obtained in our study are consistent with an effective establishment of infection, and our results point toward further investigation of the possibility of modifying host immunological factors to produce a safer antiparasitic treatment profile.

Line 178: How was the Bz administered orally: in the water or per os?

Bz was administered by gavage. This detail has been included in the revised manuscript in line 184.

Line 195: What was the disruption tissue used?

The left ventricles were isolated and halved, and one half was fixed in 4% formaldehyde in 0.1 M phosphate-buffered saline (PBS; pH 7.3) for histological analysis and the other half was homogenized using a Heidolf homogenizer (Heidolf RZR 2050, Heidolph Instruments*© GmbH & Co. KG).This description has been included in the revised manuscript. Please refer to lines 189-193.

Line 224: Histology – please inform from what chamber the heart sections were obtained and if it was the same in the mice of all groups.

As indicated in line 189, the chamber analyzed corresponded to the left ventricle.

**Reviewer #3:** -Does the analysis presented match the analysis plan? YES
-Are the results clearly and completely presented? YES, BUT DESERVES SOME CONSIDERATIONS
-Are the figures (Tables, Images) of sufficient quality for clarity? YES, BUT LEGENDS NEED IMPROVEMENTS

Line 267: Fig 1 A. Parasitemia in WT x FPR2 -/- mice infected with Trypanosoma cruzi (Dm28c): Please highlight the 2 peaks, higher at the 2nd at 27th dpi. The parasitemia curves are extremely similar, and only one time point out of 18 shows a statistically difference between the two models. I suggest the authors to be cautious with the conclusion that FPR2 -/- model has lower parasitemia than WT. One single time point and no information of a similar difference in different experiments does not allow such a conclusion. The sentence “but the overall kinetics during the acute phase was similar to that of the WT mice” is not sufficient.

Thank you very much for this comment. We agree that the difference in parasitemia observed on a single day does not allow for generalization of the same difference to the entire observation period. Therefore, the abovementioned sentence has been deleted. Likewise, the corresponding paragraph in the discussion section has been modified for consistency (please see lines 273-275 and 455-4660).

Lines 298 and 318: In Figs 2 and 3 the legend should be changed. As it is written it seems that AT RvD1 and/or Bz treatment were performed at 60 days post-infection, but in methods it is clearly stated that treatment was conduced from dpi 40 to 60 and immune and histological analysis were done at dpi 60. I suggest: “Fig 2. Serum cytokine levels at 60 days post-infection in mice infected with Trypanosoma cruzi and treated with AT RvD1 and/or Bz in the 20 precedent days.” and a similar format for Fig. 3.

We appreciate this comment, and the titles and legends of Figures 2 and 3 have been modified accordingly to include a clearer description of the experiment.

Lines 338, 369, 386, and 408: In Figures 3,5,6, and 7, authors stress “in C57BL/6 chronically infected with Trypanosoma cruzi.” Concerning the limitations of the model as a “chronic” one, I suggest that the legends state “of C57BL/6 mice infected for 60 days with Trypanosoma cruzi.”

We agree with the comments of both reviewers 2 and 3 that it is necessary to clearly specify the model used in this study. Therefore, the title has been modified and the changes suggested by the reviewer in the legends of specific figures have also been made.

**Reviewer #3:** This paper studies the immunomodulatory experimental effects of AT-RvD1 and BZ treatment in a murine model of Chagas disease, isolated and in combination. The most relevant results are the regulation of the inflammatory response both at the systemic level and in the cardiac tissue, and the reduction in cellular infiltrates, cardiomyocyte hypertrophy, fibrosis, and the parasite load in the heart tissue. The work deserves to be published, but some important issues and limitations should be addressed, especially the following concepts that need to be revised: 3 main points should be addressed:

1) the limitation of the model as a "chronic" murine model simiilar to CCC

The revised manuscript emphasizes that the study was performed on an early chronic model of *T. cruzi* infection, as mentioned above.

2) the difference of just one point in parasitemia curves between WT and FPR2 -/- infected mice

This correction has been incorporated in the revised manuscript, as mentioned above.

3) informations related to BZ treatment limitations

The limitations of the model as well as relevant references corresponding to the efficacy of Bz in preventing the chronic complications of Chagas disease have been incorporated in the revised manuscript, as mentioned above.
